# Effects of Dietary Threonine Levels on Intestinal Immunity and Antioxidant Capacity Based on Cecal Metabolites and Transcription Sequencing of Broiler

**DOI:** 10.3390/ani9100739

**Published:** 2019-09-28

**Authors:** Shuyun Ji, Xi Qi, Shuxue Ma, Xing Liu, Yuna Min

**Affiliations:** College of Animal Science and Technology, Northwest A&F University, Yangling, Shaanxi 712100, China; xinongjishu@163.com (S.J.); qixi1994314@iCloud.com (X.Q.); 18649362072@163.com (S.M.); 18829355190@139.com (X.L.)

**Keywords:** threonine, broiler, metabolism, antioxidant capacity, immunity

## Abstract

**Simple Summary:**

Threonine (Thr), an indispensable amino acid for animals and the third limiting amino acid of broilers, plays a vital role in the synthesis of gut mucosal proteins, which also has better effects on growth performance, biochemical indexes, antioxidant function, and gut morphology, as well as acting as a nutrient immunomodulator that affects the intestinal barrier function of broilers. However, it is not clear how it works in depth. The objective of the current study was to investigate the mechanism of effects of different dietary threonine levels on the antioxidant and immune capacity of broilers. Our findings suggest that a Thr level of 125% NRC (*Nutrient Requirements of Poultry*, 1994) recommendations had better effects on antioxidant and immune capacity, including resisting viruses and decreasing the abnormal proliferation of cells. As well as this, it also had better effects on maintaining the homeostasis of the body.

**Abstract:**

This study aimed to determine the effects of different dietary threonine levels on the antioxidant and immune capacity and the immunity of broilers. A total of 432 one-day-old Arbor Acres (AA) broilers were randomly assigned to 4 groups, each with 6 replicates of 18 broilers. The amount of dietary threonine in the four treatments reached 85%, 100%, 125%, and 150% of the NRC (*Nutrient Requirements of Poultry*, 1994) recommendation for broilers (marked as THR85, THR100, THR125, and THR150). After 42 days of feeding, the cecum contents and jejunum mucosa were collected for metabolic analysis and transcriptional sequencing. The results indicated that under the condition of regular and non-disease growth of broilers, compared with that of the THR85 and THR150 groups, the metabolic profile of the THR125 group was significantly higher than that of the standard requirement group. Compared with the THR100 group, the THR125 group improved antioxidant ability and immunity of broilers and enhanced the ability of resisting viruses. The antioxidant gene CAT was upregulated. PLCD1, which is involved in immune signal transduction and plays a role in cancer suppression, was also upregulated. Carcinogenic or indirect genes PKM2, ACY1, HK2, and TBXA2 were down-regulated. The genes GPT2, glude2, and G6PC, which played an important role in maintaining homeostasis, were up-regulated. Therefore, the present study suggests that 125% of the NRC recommendations for Thr level had better effects on antioxidant and immune capacity, as well as maintaining the homeostasis of the body.

## 1. Introduction

Threonine (Thr)—the third limiting amino acid in broilers after lysine and some sulfur-containing amino acids in which chickens themselves cannot synthesize [1]—is involved in many metabolic processes, such as protein synthesis and degradation, the conversion of ammonia nitrogen, and the conversion of the carbon skeleton to glucose and energy, among other processes. [2]. However, for current poultry breeds, some publications have challenged the NRC (*Nutrient Requirements of Poultry,* 1994) recommendations for amino acids as being inadequate [3,4]. Part of the reason for needing to correct the NRC in time is the adaptive change of chickens in unstable environments, the change of nutrient transport and composition, and scientific feed management. Among the four primary livestock species, the poultry publication is the oldest revised edition of the NRC [3]. A number of studies have shown that adding a higher level of Thr in broiler diet than is recommended by the NRC improved antioxidant ability and immune function [5,6,7,8,9]. Debnath et al. [10] found serum glutathione peroxidase (GSH-PX) and catalase concentration and superoxide dismutase (SOD) level increased linearly with the increasing of Thr level, which was similar to Azzam et al. [9] and Liu et al. [7]. Thr may be a nutritional immunomodulator affecting intestinal barrier function, which can improve immunity and intestinal health of broilers, especially 125% NRC Thr level [11]. The imbalance of Thr has a significant effect on the activities of glutamic oxaloacetic transaminase (GOT) and glutamic pyruvic transaminase (GPT) in poultry serum [12]. Moreira et al. [13] suggested that Thr promoted intestinal health in broilers infected with *Salmonella enteritidis*. Moderately excessive levels of Thr could reduce the incidence of *Eimeria* infestation in broilers and improve the resistance in vivo [14]. Thr could reduce the number of colonies of *Escherichia coli* and *Salmonella* in the cecum, which might be related to the increase in intestinal mucosal protein synthesis and immunoglobulin secretion caused by Thr [15]. An adequate dietary level of Thr has been shown to enhance intestinal integrity in poultry [16,17].

Our team’s previous study has shown that 125% of the NRC recommended [18] Thr level had better effects on growth performance, biochemical indexes, antioxidant function, and gut morphology of broilers [11]. However, little data focus on the further mechanism of Thr on broilers. Therefore, the present study in which metabonomics were used to identify group differential metabolites (the differential metabolites are closely related to amino acid metabolism, but few studies exist on the effect of excessive Thr on metabolites) and to associate transcriptional histology data, was undertaken to explain the in-depth mechanism of dietary Thr level on the immune function and oxidation resistance of poultry.

## 2. Materials and Methods

### 2.1. Experimental Design and Diets

The experiment was carried out under normal conditions in a layered cage with a concentration gradient in the amount of Thr, avoiding stress. The single factor complete random design was used in the experiment. Four levels of Thr were added to the basal diet. The levels of Thr in the diet were 85%, 100%, 125%, and 150% of the total Thr requirement of broilers, respectively. They were recorded in turn as THR85, THR100, THR125, and THR150. Four-hundred and thirty-two healthy Arbor Acres (AA) broilers of similar body weight were randomly divided into 4 groups with 6 replicates in each group and 18 chickens in each replicate. The experimental period lasted 42 days. The research techniques used on the living animals met the guidelines approved by the Institutional Animal Care and Use Committee (IACUC). The experimental diet was prepared according to AA broiler feeding requirements and the NRC standard. The basal diet was designed to provide all the nutrients except Thr, and its dietary composition and nutrient levels are listed in Table 1. The mass fraction of Thr in the basal diet was 0.69% and 0.62% in the early and late stages, respectively. All animal procedures were performed in accordance with the Guidelines for Care and Use of Laboratory Animals of Northwest A&F University and were approved by the Animal Ethics Committee of Northwest A&F University (NWAFU-314020038).

### 2.2. Sample Collection and Preparations

After reaching 6 weeks of age, and after fasting for 12 h, one bird from each replicate was chosen according to average body weight and slaughtered. The cecum contents were divided into four tubes and cryopreserved at −20 °C, and 24 cecum content samples were collected and measured (Wuhan Anlong Kexun Technology Co., Ltd., Wuhan, China). A comprehensive report of the metabolites in the cecum samples was obtained.

The jejunum mucosa was washed with normal saline, divided into four tubes, and cryopreserved at −20 °C. A total of 24 jejunal mucosa samples were obtained. The total RNA of the jejunum tissue was extracted, and three of the several samples from THR85-THR150 were randomly selected and assessed in terms of the quality of total RNA, which was extracted with a QIAGEN kit(Beijing Novosource Science and Technology Co., Ltd., Beijing, China) (all samples OD 260/280 should be between 1.8–2.2; the amount of all samples should be greater than 3 μg), and the degree of degradation and contamination of the RNA was measured by 1% agarose gel electrophoresis for detection (agarose imaging should be satisfied in no less than three bands: 28 S, 18 S, and 5 S; the brightness of 28 S is more than twice that of 18 S). The sequence quantity and quality of the constructed library were evaluated, then the effects of Thr on antioxidant and immune activity were studied.

### 2.3. Sample Analysis

The analysis of the cecum contents is herein briefly explained. Ultrasonic extraction and centrifugation were undertaken after the sample was dissolved in pure water and then filtered. The filtrate was then extracted. Finally, 50 μL 3-(Trimethylsilyl) propanesulfonic acid (DSS)standard solution (Anachro, Toronto, Canada) was added. The upper layer of the liquid was extracted, and the spectra were collected.

The analysis of the jejunum mucosa is briefly explained here. RNA was extracted according to the specific process in the Total RNA Kit I (Takara Bio Inc., Tokyo, Japan). First, RNA quantification and qualification were evaluated. The RNA degradation and contamination were monitored on 1% agarose gels. The RNA purity was checked using a NanoPhotometer spectrophotometer (IMPLEN, Westlake Village, CA, USA). The RNA concentration was measured using a Qubit RNA assay kit in a Qubit 2.0 fluorometer (Life Technologies, Carlsbad, CA, USA). Second, the library was prepared for transcriptome sequencing. Finally, clustering and sequencing were determined.

### 2.4. Data Analysis

First, we discuss the process of data analysis for the cecum content. The free induction decay (FID) signal was imported into Chenomx nuclear magnetic resonance (NMR) suite software to achieve 1H NMR analysis, and the Fourier transformation, phase correction, and baseline realignment were conducted automatically. The concentration and spectrum area of the peak at 0.0 ppm were defined as the standard, and the names and corresponding concentrations of 45 metabolites were obtained. The information on the intestinal content sample was included in the variable matrix as the source data for partial least squares discrimination analysis (PLS-DA), and the Ggplot 2 package was used to visualize the data analysis.

Next, we introduced the process of data analysis for the jejunum mucosa. Illumina HiSeq 2000 (Illumina, Inc., San Diego, CA, USA) was used to sequence the two ends of the sample, and the base was connected to read the sequence. The original sequence was filtered, and a high-quality sequence was obtained. The genomic localization results of all reads were combined, and the reads were assembled with the help of Cufflinks. Twelve intestinal mucosal tissues were compared and annotated using Tophat (v2.0.13) (UC, California, USA) On the basis of the results of the Tophat reference genome alignment, Cufflinks (v2.2.0) (UC, California, USA) was used to obtain the spliced transcripts for each biological replicate. Cuffmerge was used to calculate the expression of the transcripts and analyze the differential expression. The alignment sequence (mapped reads) and the spliced transcripts were submitted to Cuffdiff to search for the differentially expressed genes to complete the analysis on biological function. Finally, sequencing results were verified by qPCR.

The mRNA was extracted before cryopreservation, and the concentration of total RNA was determined by Nano-200 using 1 μg of RNA as the template. The first cDNA chain was synthesized by reverse transcription with a Super RT cDNA synthesis kit (CWBIO, Jiangsu, China). Using reverse transcriptase as a template, a matching fluorescent quantitative reagent was used to configure the sample system for analysis. Fluorescence quantitative PCR was performed using a kit. By observing the data and the final result of the real-time display on the instrument, we were able to analyze and compare the differences in gene expression. The reverse transcription reaction (20 μL) was conducted in a mixture containing 10 μL of 2 × UltraSYBR mixture (High ROX), 0.4 μL of forward primer (10 μM), 0.4 μL of reverse primer (10 μM), 1 μL of cDNA, and 8.2 μL of ddH_2_O. The primer sequences used for PCR and their gene bank accession numbers are listed in Table 2. The fluorescence quantitative PCR reaction was carried out under the conditions in a Bio-Rad iQTM5 Thermal Cycler (Bio-Rad Lab., Calif., Philadelphia, PA, USA).

## 3. Results

### 3.1. Searching for Differential Metabolites

**Figure 1:**
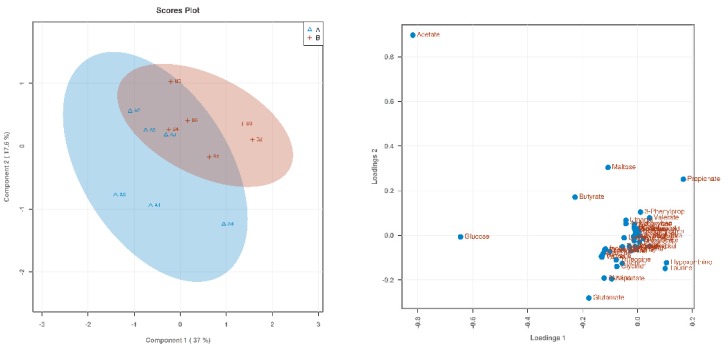
The score plot and loading plot of THR85 and THR100 from the partial least squares discrimination analysis (PLS-DA) analysis. In the above PLS-DA score chart, different groups are represented by different colors. The degree of dispersion can reflect the reproducibility of the samples and the similarity of the metabolic profiles among the sample groups. The comparison group tended to separate from each other, but the trend was not significant.

**Figure 2:**
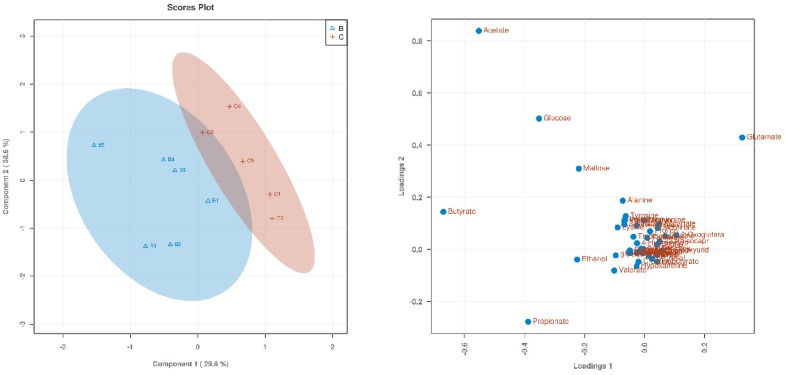
The score plot and loading plot of THR100 and THR125 from the PLS-DA analysis. The result is the picture below. The degree of separation in the score plot for this group was the most significant when compared with other groups.

**Figure 3:**
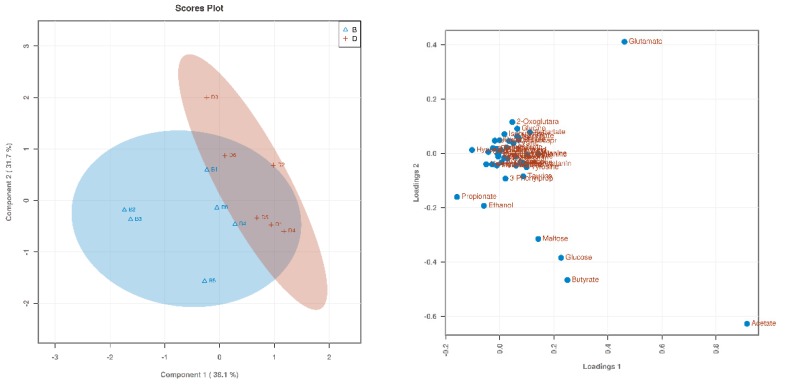
The score plot and loading plot of THR100 and THR150 from the PLS-DA analysis. In the above PLS-DA score chart, the comparison group tended to separate from each other, but the trend was not significant.

Based on 1H NMR analysis, the metabolites were identified. Through a PLS-DA analysis to find the differential metabolites, we obtained the corresponding score plots and loading plots, as shown in Figure 1, Figure 2 and Figure 3. In the score plots, the degree of dispersion can reflect the reproducibility of the samples and the similarity of the metabolic profiles among the sample groups. The results showed that the compared groups tended to be independently separated from each other. However, the group THR100 and THR125 were more distinct than the others. In the VIP diagram, differential metabolites with VIP values greater than 1 were selected (significant difference among the variables with VIP values greater than 1): glutamate, acetate, proplonate, ethanol, butyrate, and valeate (Table 3).

### 3.2. Sequencing of the Jejunal Mucosal Transcriptome

**Figure 4:**
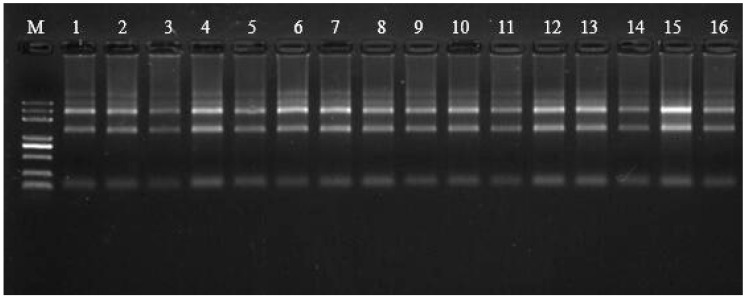
Quality report by 1% agarose gel electrophoresis. The marker is Trans 2K Plus. The figure corresponds to the following sequences: sequences 1, 2, 7, and 11 were diluted 20-fold; sequences 4, 8–10, 12–14, and 16 were diluted 10-fold; and the samples were 1 μL. Sequence 5 was diluted sevenfold, and the sample was 1 μL. Test parameters: gel concentration, 1%; voltage, 180 v; and electrophoretic time, 16 min. Agarose imaging was no less than three bands: 28 S, 18 S, and 5 S; the 28 S and 18 S bands were clear, and the brightness of 28 S was more than twice that of 18 S. The integrity of total RNA was better.

As shown in Figure 4, the total RNA extracted from the samples had good integrity, and the total RNA purity was high.

### 3.3. An Analysis of the Cross-Section of the Study Groups

On the basis of cross-group analysis, we combined the results of the metabolite data and transcriptome analysis of the mucosal samples. In the Kyoto Encyclopedia of Genes and Genomes (KEGG) enrichment analysis of all differentially expressed genes (upregulated genes and downregulated genes) between the THR100 and THR125 groups (a total of 135 pathways involving all the differentially expressed genes), all the metabolic pathways that can produce the previously mentioned six metabolites were screened, resulting in a total of 12 pathways. There were 36 differentially expressed genes, 24 were upregulated genes, and 12 downregulated genes, as follows: ABC transporters (enriched 10 differentially expressed genes), alanine, aspartate and glutamate metabolism (enriched 6 differentially expressed genes), glycolysis/gluconeogenesis (enriched 7 differentially expressed genes), nicotinate and nicotinamide metabolism (enriched 4 differentially expressed genes), 2-oxocarboxylic acid metabolism (enriched 2 differentially expressed genes), sulfur metabolism (enriched 1 differentially expressed gene), glycosaminoglycan biosynthesis–heparan sulfate/heparin (enriched 2 differentially expressed genes), biosynthesis of amino acids (enriched 4 differentially expressed genes), terpenoid backbone biosynthesis (enriched 1 differentially expressed gene), glyoxylate and dicarboxylate metabolism (enriched 1 differentially expressed gene), arginine and proline metabolism (enriched 2 differentially expressed genes), and the calcium signaling pathway (enriched 9 differentially expressed genes).

The pathways in which glutamate was involved were ABC transporters, alanine, aspartate and glutamate metabolism, nicotinamide and nicotinamide metabolism, 2-oxocarboxylic acid metabolism, biosynthesis of amino acids, glyoxylate and dicarboxylate metabolism, arginine and proline metabolism, and the calcium-signaling pathway. The codes of the pathways in the KEGG database are gga02010, gga00250, gga00760, gga01210, gga01230, gga00630, gga00330, and gga04020. The pathway in which ethanol was involved was glycolysis/gluconeogenesis. The number of the pathway in the KEGG database is gga00010. The pathways in which acetic acid was involved included sulfur metabolism, glycosaminoglycan biosynthesis–heparan sulfate/heparin, terpenoid backbone biosynthesis and glycolysis/gluconeogenesis. The corresponding codes of the pathways in the KEGG database are gga00920, gga00534, gga00900, and gga0010, respectively.

**Figure 5:**
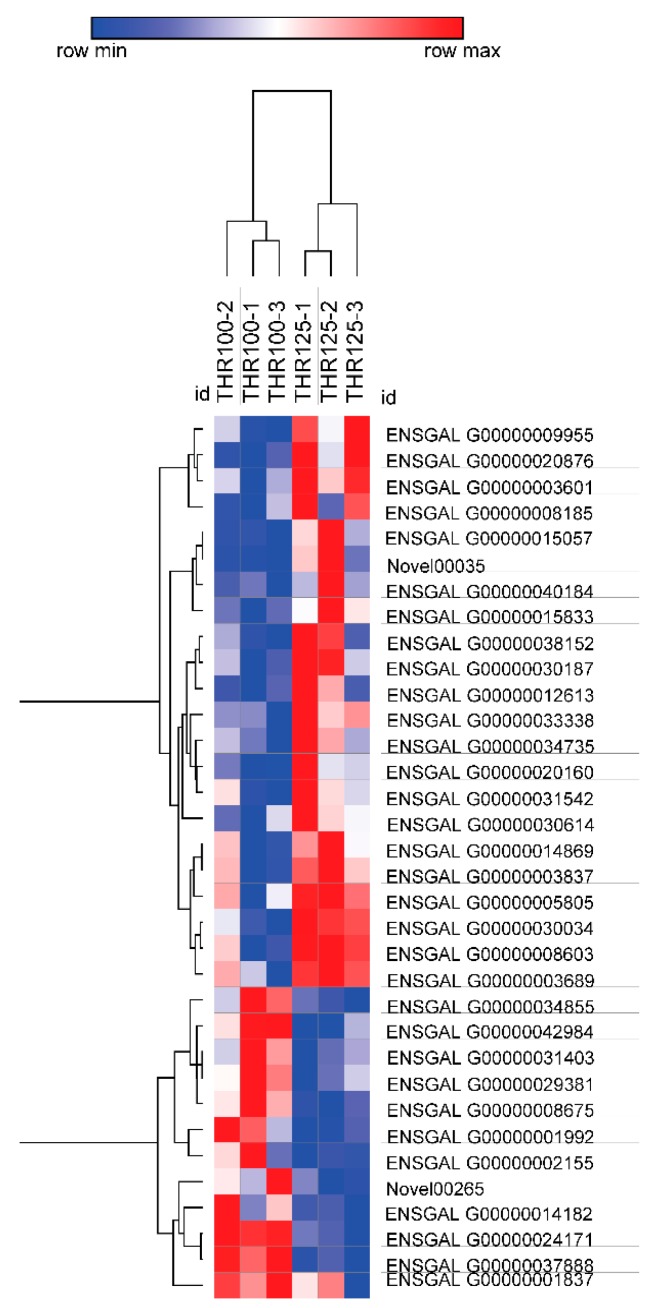
Expression profiles of differential genes between THR100 and THR125_._ The heat map showed the differential gene expression profiles in THR100 and THR125 groups. Each row represents the level of expression of each differentially expressed gene; each column represents a biological repetition. The expression level of each gene is expressed by fragments per kilobase of exon per million reads mapped (FPKM) value. The FPKM value for each differential gene was used for plotting. Red indicates that the gene is highly expressed, and blue indicates a low expression level.

Expression profiles of differential genes between THR100 and THR125 are presented in Figure 5. There were 24 up-regulated genes and 12 down-regulated genes. Of the 36 genes, eight were screened for biological significance related to immune and antioxidant regulation, among which the upregulated genes were CAT, GPT2, PLCD1, GLUD2, and G6PC, and the down-regulated genes were PKM2, HK2, and TBXA2R.

### 3.4. Identification of the Differentially Expressed Genes by qPCR

To further verify the results of transcriptome sequencing, fluorescence quantitative polymerase chain reaction (real-time PCR) was performed. We selected five important genes (ABCC1, CAT, G6PC, PLCD1, and PKM2) for verification. The results showed that the gene expression pattern of these genes was consistent with the results of transcriptome sequencing and that the common phase relationship was 0.901 (*p* < 0.01). Therefore, the results of transcriptome sequencing were effective.

## 4. Discussion

Thr is the third limiting amino acid of broilers, which plays a key role in antioxidant and immune capacity. On the basis of group cross, we discovered the reason why the dietary Thr levels at 125% and 100% of the recommended NRC had different effects. Eight of the 36 genes in 12 pathways were screened for biological significance related to immune and antioxidant regulation. The upregulated genes were CAT, GPT2, GLUD2, G6PC, and PLCD1; the downregulated genes were PKM2, HK2, and TBXA2R.

In our study, the CAT expression was upregulated; Debnath et al. [10] came to the same conclusion—of great significance to the antioxidant ability of broilers—finding it to be higher in the THR125 group when compared with THR100. In broilers, Thr plays a vital role in the synergistic effects of the scavenging of reactive oxygen free radicals and the regulation of peroxidase on membrane lipid peroxidation. Liu et al. [7] and Debnath et al. [10] found that when the addition of THR was higher than the recommended amount, the activity of serum GSH-PX and total superoxide dismutasewas higher. Our results indicated that excessive Thr could significantly increase the antioxidant enzyme activity of broilers, which is consistent with Habte et al. [12] and Azzam et al. [9]. The improved oxidation state may be due to the simultaneously enhanced immune function, reduced colonization of pathogenic bacteria, and improved intestinal health.

The expression of GPT2 was upregulated, which is involved in the metabolism of proteins and amino acids. GPT2 is involved in gluconeogenesis, which converts a variety of non-sugar substances into glucose or glycogen and assists in amino acid metabolism [19]. GPT2 is one of the two major aminotransferases (other than glutamic oxaloacetic transaminase) in poultry, and plays an important role in the maintenance of homeostasis [20]. A high activity of aminotransferase is beneficial to amino acid metabolism in vivo. Liu et al. [7] used an automatic biochemical analyzer to measure the activity of GOT and GPT, and found that dietary levels of Thr above the recommendation by the NRC could significantly increase the activities of these two enzymes, and the same conclusion was reached by Gao et al. [21] and Habte et al. [12].

PLCD1 participates in the transduction of multiple immune signals. It can promote apoptosis and inhibit the invasion of tumor cells [22]. PLCD1 encodes enzymes that regulate energy metabolism, calcium homeostasis, and intracellular signaling pathways in animals. Many scientific findings indicate that it has been identified as a tumor suppressor gene for many types of cancer, including pancreatic cancer, esophageal squamous cell carcinoma, gastric cancer, chronic myeloid leukemia, and breast cancer [23,24,25,26,27].

G6PC deficiency can cause metabolic disorders such as glycogen storage disease Ia (GSD Ia) [28], which is a key rate-limiting glycosylation gene [29]. G6PC plays an important role in maintaining blood glucose stability, which is a key enzyme for the final reaction between hepatic glycosylation and glycogen degradation in broilers [30].

In our study, the results showed that 125% Thr supplementation, some oncogenes were downregulated, tumor proliferation was inhibited, immunity and the antioxidation capacity were enhanced, and the antitumor mechanism was induced. Satomura et al. [31] and Tomas et al. [32] reached similar conclusion, respectively. The four oncogenes that were downregulated have long been proven to have carcinogenic effects in humans, and further tests are needed to confirm on the similar effects on broilers.

PKM2, which can regulate apoptosis and proliferation of cancer cells and meet their high energetic and biosynthetic demands of rapid growth and proliferation is a marker of the metabolic transition of cancer cells [33,34,35,36,37], and also regulated by many oncogenes, with its upregulation promoting tumor formation [38,39]. Yang et al. [40] reported that nuclear translocation of the glycolic key enzyme, PKM2, helps cancer cells survive under metabolic stress. PKM2 is the link between oncogenes and metabolism, which plays a central role in the metabolic recombination of tumor cells, the activation of cancer metabolism, and proliferation of cancer cells [41].

HK2 downregulation inhibits the regulatory factors of tumor metabolism. Palmieri et al. [42] pointed out that tumor metastasis was associated with high expression of HK2. Tao et al. [43] proposed that HK2 could promote tumor proliferation and survival. HK2 is highly expressed in tumor cells and is the first to use of mitochondria-synthesized HK2 catalyzes the rate-limiting and first step of glucose metabolism. HK2 is highly expressed in many cancers, including breast cancer, ovarian cancer, and colon cancer [44].

TBXA2R is usually expressed in tumors, especially in invasive tumors, which has a potential effect on the metastasis of cancer cells through its metabolite TAX2, which has previously been reported to be found in various tumors, including prostate, glioma, and melanoma [45].

In our study, one of the differential pathways was the ATP-binding cassette transporter (ABC transport). There were 10 different genes, which included 9 with an upregulated expression: ABCA1, ABCA2, ABCB1, ABCB4, ABCC2, ABCC6, ABCD2, ABCG5, ABCG8, and with a downregulated expression, ABCB3 [46]. The results of this change demonstrate the correctness of our previous experiment—compared with the 100% NRC group, the concentration of serum total protein and globulin in the 125% NRC group was significantly higher than that of Debnath et al. [10], indicating that the total protein concentration and serum globulin level increased linearly. The conversion efficiency of some proteins affects the metabolic process. Sufficient levels of Thr promote the trans-membrane transport of antibodies and biological molecules, and the synthesis of proteins. Antibodies and immunoglobulins are all proteins, and Thr is an important limiting amino acid for poultry immunoglobulin molecules. The sufficient supplying of Thr contributes to the production of immunoglobulins and lymphocytes.

The imbalance of Thr leads to deamination, and, in addition, it interferes with the absorption and utilization of other amino acids. THR85 did not result in significant differences in metabolites compared with THR100, which may be because the Thr supply can meet the basic maintenance needs of the broilers and other amino acids act as Thr by binding to certain substances. The effect of THR150 was not satisfactory. Baker et al. [47] noted that excess Thr could be converted to glycine in vivo. At the same time, an excess of Thr could also result in a relative deficiency and low utilization of other amino acids.

In different growth and developmental stages or under different conditions, the expression of genes in the same cell or tissue is different. Gene expression has temporal and spatial specificity [48]. Therefore, the evaluation of the immunity and antioxidant capacity of broilers via gene expression in intestinal tissue has some limitations, but it is of great importance to studies on the optimal content of Thr in broiler feed. In this experiment, the results on the regulation of differential genes was combined with those from previous studies, fully indicating that the level of Thr at 125% of the standard recommended amount of the NRC could significantly improve the immunity and antioxidant capacity of broilers. This concentration of Thr can decrease the abnormal proliferation of resistant cells in broilers. The antioxidant gene CAT was upregulated, along with PLCD1, which is involved in immune signal transduction and plays a role in cancer suppression. Carcinogenic or indirect genes PKM2, ACY1, HK2, TBXA2, on the other hand, were down-regulated. The genes GPT2, glude 2, and G6PC, which played an important role in maintaining homeostasis, were up-regulated.

## 5. Conclusions

Thr level of 125% NRC recommendations had better effects on antioxidant and immune capacity, including resisting viruses and decreasing the abnormal proliferation of cells. Alongside this, it also had better effects on maintaining the homeostasis of the body.

## Figures and Tables

**Table 1:** Ingredients and nutrient composition of the basal diet (as-fed basis).

Items (%)	Starter (0 to 21 d)	Finisher (22 to 42 d)
Ingredients		
Corn	31.92	32.54
Soybean meal	26.25	18.40
Wheat	25.00	30.00
Peanut meal ^1^	5.00	5.00
Soy bean oil	4.20	4.80
Distillers dried grains with solubles	3.00	5.00
Dicalcium phosphate	1.65	1.35
Limestone	1.10	1.30
Premix ^2^	1.00	1.00
L-lysine	0.32	0.28
Salt	0.25	0.25
DL-methionine	0.18	0.13
Choline chloride	0.10	0.10
Antioxidant	0.03	0.03
L-threonine	0.00	0.00
Total	100.00	100.00
Calculated composition		
Metabolizable energy (kcal/kg)	3000.00	3100.00
Total phosphorus	0.68	0.62
Available phosphorous	0.45	0.40
Calcium	0.90	0.90
Sodium chloride	0.30	0.30
Analyzed composition		
Crude protein	20.87	19.10
Lysine	1.24	1.11
Methionine	0.50	0.42
Isoleucine	0.67	0.58
Threonine ^3^	0.69	0.62
Tryptophan	0.23	0.20
Valine	0.85	0.74
Arginine	1.32	1.14

^1^ Per kilogram of peanut meal (g/kg): crude protein, 523 g; ash, 62.4 g; water, 54.5 g; aspartic acid, 65.2 g; threonine, 14.7 g; serine, 24.7 g; glutamic acid, 100.4 g; glycine, 29.2 g; alanine, 20.9 g; valine, 20.0 g; methionine, 5.6 g; isoleucine, 16.8 g; leucine, 33.6 g; tyrosine, 21.4 g; phenylalanine, 26.4 g; lysine, 16.3 g; histidine, 12.1 g; arginine, 64.3 g; and proline, 25.8 g. ^2^ Provided per kilogram of diet: vitamin A, 45000 IU; cholecalciferol, 14000 IU; vitamin E, 90 IU; vitamin K3, 10 mg; thiamin, 7.36 mg; riboflavin, 25.6 mg; pyridoxine, 19.68 mg; vitamin B12, 0.1 mg; nicotinamide, 158.4 mg; calcium pantothenate, 46 mg; folic acid, 3.325 mg; biotin, 0.7 mg; Cu, 7.25 mg; Fe, 72 mg; Zn, 74.52 mg; Mn, 71.232 mg; Se, 0.3 mg; I, 0.5 mg; Co, 0.2 mg. ^3^ The starter (0 to 21 d) poultry requirements for Thr (%) in the four groups (THR85, THE100, THR125, and THR150 in turn) are listed: 0.59%, 0.69%, 0.86%, and 1.04%; the finisher (22 to 42 d) poultry requirements for Thr (%) in the four groups (THR85, THR100, THR125, and THR150 in turn) are listed: 0.53%, 0.62%, 0.78%, and 0.9%.

**Table 2:** Nucleotide primer sequences of the PCR primers.

Gene Symbol	Ensemble Accession No.	Forward Primer (5′-3′)	Reverse Primer (5′-3′)	Product Size/bp
ABCC2	ENSGALG00000007395	TGTCCTTACCATTGCCCACC	CCACTTACATCCGCTCCACC	197
CAT	ENSGALG00000030187	GAACGCCGCATAGTAAGA	GAGGGTCACGAACAGTAT	414
PKM2	ENSGALG00000001992	ATGTCGAAGCCCCATAGTGAA	TGGGTGGTGAATCAATGTCCA	118
G6PC	ENSGALG00000030034	GCGTCTGGTATGTAATGG	AGAATAACTTGATGAGGGA	181
PLCD1	ENSGALG00000005805	CCAGTGAACGAGCCAAGAAG	TTCCACACAGCGGCAACCTT	245
GAPDH	ENSAPLG00000013511	TCTTCACCACCATGGAGAAG	CAGGACGCATTGCTGACAAT	154

**Table 3:** The VIP scores of THR100 and THR125 for partial least squares discrimination analysis (PLS-DA). ^1^

Metabolite	VIP Scores
Propionate	3.82660
Butyrate	3.58300
Glutamate	2.89960
Acetate	1.40370
Ethanol	1.28460
Valerate	1.05500
Aspartate	0.78458
Glucose	0.77806
Lysine	0.69175
2-Oxoglutarate	0.62168
Hypoxanthine	0.55743
3-Phenylpropionate	0.52454
Glycine	0.49846
Uridine	0.47948
Methionine	0.45836

^1^ There were significant differences among the metabolites whose VIP value is greater than 1. There were six metabolites with a VIP score greater than 1: glutamate, acetate, proplonate, ethanol, butyrate, and valeate.

## References

[1] Kidd M., Kerr B.J. (1996). L-threonine for poultry: A review. J. Appl. Poult. Res..

[2] Ballèvre O., Cadenhead A., Calder A.G., Rees W.D., Lobley G.E., Fuller M.F., Garlick P.J. (1990). Quantitative partition of threonine oxidation in pigs: effect of dietary threonine. Am. J. Physiol. Metab..

[3] Applegate T.J., Angel R. (2014). Nutrient requirements of poultry publication: History and need for an update. J. Appl. Poult. Res..

[4] Kidd M.T., Corzo A., Dozier W.A. (2008). Dietary Amino Acid Responses of Broiler Chickens. J. Appl. Poult. Res..

[5] Qaisrani S.N., Ahmed I., Azam F., Bibi F., Pasha T.N., Azam F. (2018). Saima Threonine in broiler diets: An updated review. Ann. Anim. Sci..

[6] Shirisha R., Umesh B.U., Prashanth K. (2018). Effect of L-Threonine supplementation on broiler chicken: A review. J. Pharm. Innov..

[7] Liu S.G., Qu Z.X., Meng G.H., Gao Y.P., Min Y.N. (2017). Effects of dietary threonine levels on growth performance, antioxidant capacities and immune function of broiler chickens. Acta Agric. Boreali Occident. Sin..

[8] Chen Y.P., Cheng Y.F., Li X.H., Yang W.L., Wen C., Zhuang S., Zhou Y.M. (2016). Effects of threonine supplementation on the growth performance, immunity, oxidative status, intestinal integrity, and barrier function of broilers at the early age. Poult. Sci..

[9] Azzam M., Dong X., Xie P., Zou X., Azzam M. (2012). Influence of L–threonine supplementation on goblet cell numbers, histological structure and antioxidant enzyme activities of laying hens reared in a hot and humid climate. Br. Poult. Sci..

[10] Debnath B.C. (2019). Effects of Supplemental Threonine on Antioxidant Enzyme Activities and Haemato-biochemical Profile of Commercial Broilers in Sub-Tropics. J. Anim. Res..

[11] Min Y.N., Liu S.G., Qu Z.X., Meng G.H., Gao Y.P. (2017). Effects of dietary threonine levels on growth performance, serum biochemical indexes, antioxidant capacities, and gut morphology in broiler chickens. Poult. Sci..

[12] Habte-Tsion H.M., Ge X., Liu B., Xie J., Ren M., Zhou Q., Miao L., Pan L., Chen R. (2015). A deficiency or an excess of dietary threonine level affects weight gain, enzyme activity, immune response and immune-related gene expression in juvenile blunt snout bream (Megalobrama amblycephala). Fish Shellfish Immunol..

[13] Filho A.L.D., Oliveira C.J.B., Neto O.C.F., De Leon C.M., Saraiva M.M.S., Andrade M.F.S., White B., Givisiez P.E.N. (2018). Intra-Amnionic Threonine Administered to Chicken Embryos Reduces Salmonella Enteritidis Cecal Counts and Improves Posthatch Intestinal Development. J. Immunol. Res..

[14] Wils-Plotz E.L., Jenkins M.C., Dilger R.N. (2013). Modulation of the intestinal environment, innate immune response, and barrier function by dietary threonine and purified fiber during a coccidiosis challenge in broiler chicks. Poult. Sci..

[15] (2006). Specific Amino Acids Increase Mucin Synthesis and Microbiota in Dextran Sulfate Sodium–Treated Rats. J. Nutr..

[16] Adedokun S.A., Olojede O.C. (2019). Optimizing Gastrointestinal Integrity in Poultry: The Role of Nutrients and Feed Additives. Front. Veter. Sci..

[17] Dong X.Y., Azzam M.M.M., Zou X.T. (2017). Effects of dietary threonine supplementation on intestinal barrier function and gut microbiota of laying hens. Poult. Sci..

[18] NRC (1994). Nutrient Requirements of Poultry.

[19] Hicks J.A., Porter T.E., Sunny N.E., Liu H.C. (2019). Delayed Feeding Alters Transcriptional and Post-Transcriptional Regulation of Hepatic Metabolic Pathways in Peri-Hatch Broiler Chicks. Genes.

[20] Yuan C., Song H.H., Jiang Y.J., Azzam M., Zhu S., Zou X.T. (2013). Effects of lead contamination in feed on laying performance, lead retention of organs and eggs, protein metabolism, and hormone levels of laying hens. J. Appl. Poult. Res..

[21] Gao Y.J., Yang H.J., Liu Y.J., Chen S.J., Guo D.Q., Yu Y.Y., Tian L.X. (2014). Effects of graded levels of threonine on growth performance, biochemical parameters and intestine morphology of juvenile grass carp Ctenopharyngodon idella. Aquaculture.

[22] Mu H., Wang N., Zhao L. (2015). Methylation of PLCDl and adenovirus- mediated PLCD1 overexpression elicits a gene therapy effect on human breast cancer. Exp. Cell Res..

[23] Carvou N., Norden A.G., Unwin R.J., Cockcroft S. (2007). Signalling through phospholipase C interferes with clathrin-nediated endocytosis. J. Cell Signal.

[24] Hu D., Jiang Z. (2016). Phospholipase Cδ1 (PLCD1) inhibits the proliferation, invasion and migration of CAPAN-1 and BXPC-3 pancreatic cancer cells. J. Cell Mol. Immun..

[25] Hu X.T., Zhang F.B., Fan Y.C., Shu X.S., Wong A.H.Y., Zhou W., Shi Q.L., Tang H.M., Fu L., Guan X.Y. (2009). Phospholipase C delta 1 is a novel 3p22.3 tumor suppressor involved in cytoskeleton organization, with its epigenetic silencing correlated with high-stage gastric cancer. Oncogene.

[26] Chen J.B., Song J.J., Liu Q., Li Y., Yang Z.S., Yang L., Xiang T.X., Ren G.S. (2012). Epigenetic inactivation of PLCD1 in chronic myeloid leukemia. Int. J. Mol. Med..

[27] Xiang T.X., Li L.L., Fan Y.C., Jiang Y.Y., Ying Y., Putti T.C., Tao Q., Ren G.S. (2010). PLCDl is a functional tumor suppressor inducing G2/M arrest and frequently methylated in breast cancer. J. Cancer Biol. Ther..

[28] Roseman D.S., Khan T., Rajas F., Jun L.S., Asrani K.H., Isaacs C., Farelli J.D., Subramanian R.R. (2018). G6PC mRNA Therapy Positively Regulates Fasting Blood Glucose and Decreases Liver Abnormalities in a Mouse Model of Glycogen Storage Disease 1a. Mol. Ther..

[29] Sen S., Das C. (2018). Managing the sugar factory: New feather in the cap for nuclear factor Y. J. Biol. Chem..

[30] Zhang N., Geng T., Wang Z., Zhang R., Cao T., Camporez J.P., Cai S.Y., Liu Y., Dandolo L., Shulman G.I. (2018). Elevated hepatic expression of H19 long noncoding RNA contributes to diabetic hyperglycemia. JCI Insight.

[31] Satomura K., Tobiume S., Tokuyama R., Yamasaki Y., Kudoh K., Maeda E., Nagayama M. (2007). Melatonin at phar-macological doses enhances human osteoblastic differentiation in vitro and promotes mouse cortical bone formation in vivo. J. Pineal Res..

[32] Tomas C., Montes A.C. (2005). A proposed mechanism to explain the stimulatory effect of melatonin on antioxidantive en-zymes. J. Pineal Res..

[33] Amin S., Yang P., Li Z. (2019). Pyruvate kinase M2: A multifarious enzyme in non-canonical localization to promote cancer progression. Biochim. Biophys. Acta (BBA) Bioenerg..

[34] Nicholas W., Melo J.D., Tang D. (2013). PKM2, a Central Point of Regulation in Cancer Metabolism. Int. J. Cell. Biol..

[35] Lee J., Kim H.K., Han Y.M., Kim J. (2008). Pyruvate kinase isozyme type M2 (PKM2) interacts and cooperates with Oct-4 in regulating transcription. Int. J. Biochem. Cell Boil..

[36] Hoshino A., Hirst J.A., Fujii H. (2007). Regulation of Cell Proliferation by Interleukin-3-induced Nuclear Translocation of Pyruvate Kinase. J. Biol. Chem..

[37] Stetak A., Veress R., Ovádi J., Csermely P., Ullrich A., Kéri G. (2007). Nuclear Translocation of the Tumor Marker Pyruvate Kinase M2 Induces Programmed Cell Death. Cancer Res..

[38] Desai S., Ding M., Wang B., Lu Z., Zhao Q., Shaw K. (2014). Tissue-specific isoform switch and DNA hypomethylation of the pyruvate kinase PKM gene in human cancers. Oncotarget.

[39] Yang W., Xia Y., Ji H., Zheng Y., Liang J., Huang W., Gao X., Aldape K., Lu Z. (2011). Nuclear PKM2 regulates β-catenin transactivation upon EGFR activation. Nature.

[40] Yang Y.C., Chien M.H., Hsiao M. (2018). Glucose limited enhance the cancer stem cell population through PKM2/AMPK-dependent signaling. FASEB J..

[41] Wu S.F. (2013). Dual roles of PKM2 in cancer metabolism. Acta Bioch. Bioph. Sin..

[42] Palmieri D., Fitzgerald D., Shreeve S.M., Hua E., Bronder J.L., Weil R.J., Davis S., Stark A.M., Merino M.J., Kurek R. (2009). Analyses of resected human brain metastases of breast cancer reveal the association between up-regulation of hexokinase 2 and poor prognosis. Mol. Cancer Res..

[43] Tao T., Shen Z., Xiang P., Hunag T., Xuan Q. (2017). Expression of hk2 gene in prostate cancer and its effect on malignant phenotype of prostate cancer cells. Int. J. Clin. Exp. Pathol..

[44] Gareth W., Anthony D.J., Robert E.M., Jiang W.G. (2005). TBXAS1 and the thromboxane A2 receptor, TBXA2R, in human breast cancer. BioMed Cent..

[45] Borths E.L., Locher K.P., Lee A.T., Rees D.C. (2002). The structure of Escherichia coli BtuF and binding to its cognate ATP binding cassette transporter. Proc. Natl. Acad. Sci. USA.

[46] Patra K.C., Wang Q., Bhaskar P.T., Miller L., Wang Z., Wheaton W., Chandel N., Laakso M., Muller W.J., Allen E.L. (2013). Hexokinase 2 Is Required for Tumor Initiation and Maintenance and Its Systemic Deletion Is Therapeutic in Mouse Models of Cancer. Cancer Cell.

[47] Baker D.H., Hill T.M., Kleiss A.J. (1972). Nutritional Evidence Concerning Formation of Glycine from Threonine in the Chick. J. Anim. Sci..

[48] Wang Z., Gerstein M., Snyder M. (2009). RNA-Seq: A revolutionary tool for transcriptomics. Nat. Rev. Genet..

